# Structural inventory of cotranslational protein folding by the eukaryotic RAC complex

**DOI:** 10.1038/s41594-023-00973-1

**Published:** 2023-04-20

**Authors:** Miglė Kišonaitė, Klemens Wild, Karine Lapouge, Genís Valentín Gesé, Nikola Kellner, Ed Hurt, Irmgard Sinning

**Affiliations:** Heidelberg University Biochemistry Center (BZH), Heidelberg, Germany

**Keywords:** Cryoelectron microscopy, Chaperones

## Abstract

The challenge of nascent chain folding at the ribosome is met by the conserved ribosome-associated complex (RAC), which forms a chaperone triad with the Hsp70 protein Ssb in fungi, and consists of the non-canonical Hsp70 Ssz1 and the J domain protein Zuotin (Zuo1). Here we determine cryo-EM structures of *Chaetomium thermophilum* RAC bound to 80S ribosomes. RAC adopts two distinct conformations accommodating continuous ribosomal rotation by a flexible lever arm. It is held together by a tight interaction between the Ssz1 substrate-binding domain and the Zuo1 N terminus, and additional contacts between the Ssz1 nucleotide-binding domain and Zuo1 J- and Zuo1 homology domains, which form a rigid unit. The Zuo1 HPD motif conserved in J-proteins is masked in a non-canonical interaction by the Ssz1 nucleotide-binding domain, and allows the positioning of Ssb for activation by Zuo1. Overall, we provide the basis for understanding how RAC cooperates with Ssb in a dynamic nascent chain interaction and protein folding.

## Abstract (web summary)

Here, the authors use cryo-EM to study cotranslational protein folding by the eukaryotic RAC complex and its conformational dynamics on the 80S ribosome

## Main

**Fig. 1:**
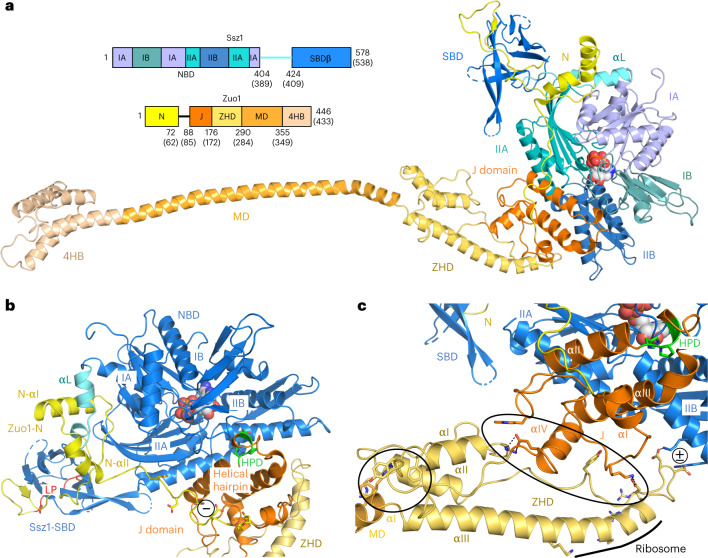
Architecture of full-length RAC reveals new contacts between Ssz1 and Zuo1. **a**, Cryo-EM structure of *C. thermophilum* RAC in ribbon representation and its domain architecture (residue numbers are given for *C. thermophilum*, with the corresponding residues in *S. cerevisiae* in parentheses). For the purpose of representation, only the RAC-1 conformation is shown. Ssz1 comprises an NBD (shades of blue), a linker (αL; cyan) and SBD-β (dark blue). NBD lobes IA, IIA, IB and IIB are shown in different shades of blue. Zuo1 comprises an N-terminal domain (N; yellow), J domain (J; orange), ZHD (pale yellow), MD (pale orange) and four-helix bundle (4HB; tan). Disordered residues are indicated as dashed lines. ATP is shown in sphere representation. **b**, The Ssz1-Zuo1N interface is enlarged by an extension of Zuo1N-αI and Zuo1N-αII. Zuo1-J shows the canonical J domain fold with a central helical hairpin and contacts Ssz1-NBD. It bridges lobes IIA to IIB and contains the conserved HPD motif (*Ct*Zuo1 His133-Pro134-Asp135; green). The HPD motif breaks the first helix at its C terminus and is completely masked by its Ssz1-NBD interaction. Negative charges in the Zuo1N-J linker are indicated by ‘−’ in a circle; LP-motif binding to the Ssz1-SBD is highlighted in red. **c**, Zuo1-J and -ZHD are directly linked and form a rigid entity. The J-ZHD contact involves salt bridges and stacking aromates (large black ellipse). An additional small contact (present only in the RAC-1 conformation) between Zuo1-ZHD and Ssz1-NBD involves two aspartates adjacent to ZHD-αIII (annotated by ‘±’ in a circle). The Zuo1 ZHD-MD contact is indicated by a small black circle, where a tight *π*–cation stacking network fixes the first two turns of MD-αI to the ZHD.

Efficient protein folding is a challenge for proteostasis in all organisms; during translation this is already ensured by ribosome-associated chaperones, which modulate protein synthesis and are among the first contacts of the emerging polypeptides^1,2^. The ribosome-associated complex (RAC) is conserved in eukaryotes, and in *Saccharomyces cerevisiae* comprises a stable heterodimer formed by the non-canonical Hsp70 homolog Ssz1 and the J domain protein (JDP) Zuo1^3,4^. Ssz1 differs from canonical Hps70s in several ways: it binds adenosine triphosphate (ATP) but does not hydrolyze, and ATP binding is not required for its function^5^; it has a unique domain arrangement and a truncated substrate-binding domain (SBD) with only a rudimentary β-sandwich domain (SBD-β); it lacks the α-helical lid domain (SBD-α) and the conserved linker^3,6^, which is central to the allosteric regulation of canonical Hsp70 activity^7^. Instead, the linker in Ssz1 is extended and adopts an αβ-structure that intertwines with the Zuo1 N terminus, which complements SBD-β and molds this unusual Hsp70/JDP pair into a stable, functional unit^6,8^ (Fig. 1a). Zuo1 is a class C JDP and the only Hsp40 that activates the ribosome-associated Hsp70 protein Ssb (encoded by two isoforms, *SSB1* and *SSB2*, that are nearly identical)^3,9^. In general, JDPs play a central role in specifying and directing Hsp70 functions^10–12^. All JDPs, including Zuo1, comprise a universally conserved HPD (histidine-proline-aspartate) motif, which is essential for stimulating ATPase activity in JDP/Hsp70 pairs^13^. The co-chaperone function of Zuo1 requires the presence of Ssz1^4^ and results in RAC and Ssb forming a functional chaperone triad at the ribosome^14,15^. Nascent chain (NC) binding by Ssb requires the presence of RAC^16^ and accelerates translation^17^. Both RAC proteins contact the NCs and form a relay system that transfers polypeptides from Zuo1 via Ssz1 to Ssb^8^. The majority of nascent proteins interact with Ssb by multiple binding–release cycles^18^. RAC binding to the ribosome has been thoroughly studied by crosslinking experiments and low-resolution cryo-EM structures, indicating flexible conformations on idle 80S ribosomes and interactions with both the 40S and 60S subunits^5,19–24^. However, the integration of Ssb, its ATPase cycle, NC and ribosome interactions into the workings of RAC has remained incomplete. Thus, a complete model of RAC alone as well as in the context of the ribosome could not be established. A recent in vivo crosslinking study suggests a pathway of Ssb movement at the ribosome and places Ssb next to the Ssz1 nucleotide-binding domain (NBD)^24^. However, all these data did not provide a clear and complete picture of the RAC/Ssb triad at the ribosome, and the molecular mechanisms of this unique chaperone relay system were not resolved. In this Article, we fill these gaps by describing RAC in two conformations bound to 80S ribosome–nascent chain complexes (RNCs), a complete description of RAC and its ribosome contacts, and with a structure-based model for Ssb activation by RAC at the ribosome.

## Results

### Complete RAC reveals contacts between Ssz1-NBD and Zuo1 J-ZHD

**Extended Data Fig. 1:**
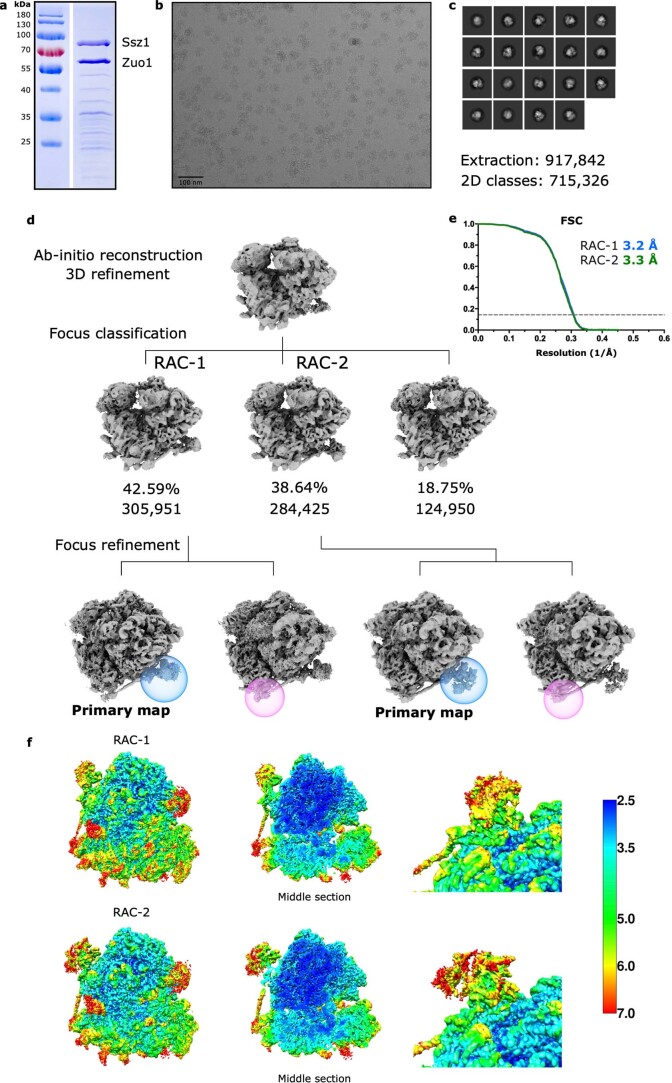
Sample preparation of *C. thermophilum* RAC-80S and processing of the cryo-EM data. **a**, Purified sample from *C. thermophilum* Ssz1 pullout analyzed by SDS-PAGE and Coomassie staining. The analysis shows prominent bands of Ssz1, Zuo1 and also 80S ribosomal proteins **b**, Cryo-EM micrograph of the RAC-80S ribosome complexes. A total of 6,662 micrographs were collected on a Titan Krios 300 kV microscope and subjected to beam-induced motion correction. **c-d**, 2D classes and a flow chart showing the stages of cryo-EM image processing. A total of 917,842 particles were auto-picked and after multiple rounds of 2D classification resulted in a selection of 715,326 particles. The particles were subjected to 3D refinement and 3D classification centered on RAC. Focus refinement was performed with masks as shown by blue and pink spheres. Data processing was done using RELION 3.1 and cisTEM **e**, The 0.143 FSC cut-off criteria indicates that the cryo-EM maps of the RAC-80S ribosome in RAC-1 and RAC-2 conformations have average resolutions of 3.2 Å and 3.3 Å, respectively. **f**, Local resolution distribution displayed on the primary cryo-EM density maps of the RAC-1 and RAC-2 complexes and close up view of RAC (the color bar shows local resolution in Å). Source data

**Extended Data Fig. 2:**
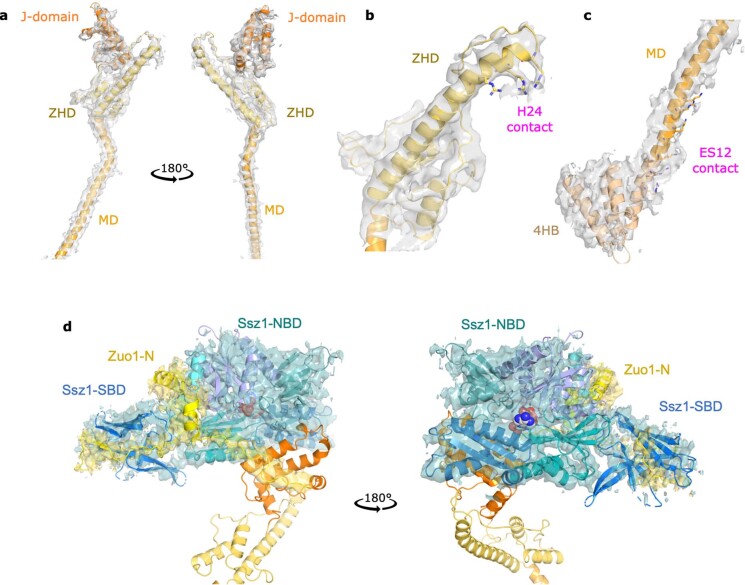
Segmented cryo-EM map of RAC (from RAC-1 conformation), superimposed with the atomic model. **a**, Density map and model of Zuo1 J domain, ZHD and MD. **b**, Close view of ZHD domain, with C1 contact (Arg253, 257, and 261) shown in sticks. **c**, Close view of MD and 4HD domains, with C2 contact (Arg362, 365 and Lys350, 354, 358, 359, 369) shown in sticks. **d**, Density map of Ssz1 (blue) and Zuo1 N-terminal domain (yellow) and corresponding models. Domains are colored as in Fig. 1. Density maps for panels **a, b** and **d** are from the primary map, while the density map for panel **c** is from RAC-40 area-focused map.

**Extended Data Fig. 3:**
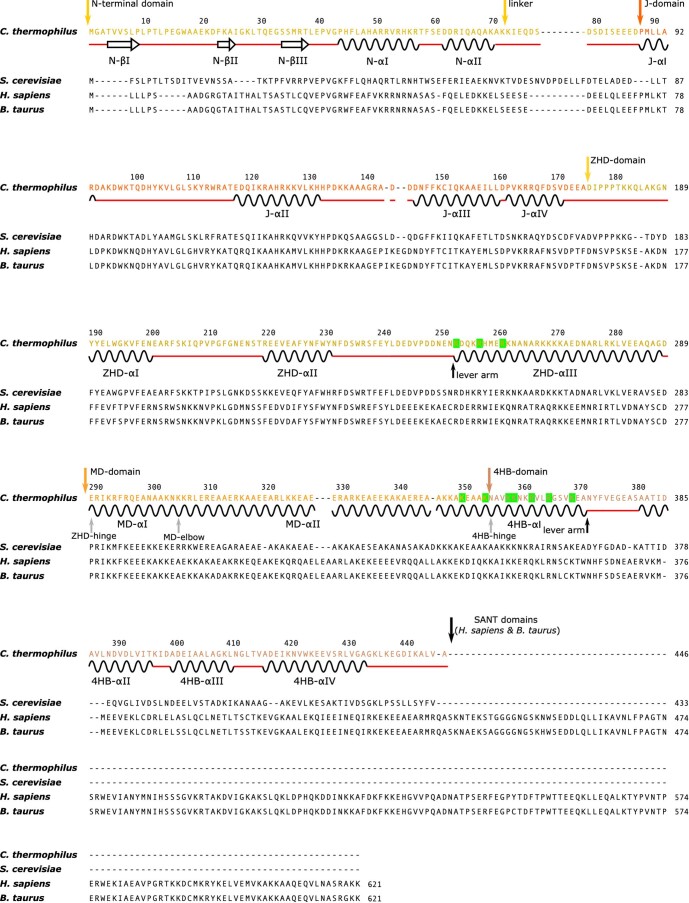
Multiple sequence alignment and secondary structure of *C. thermophilum* Zuo1. Sequence alignment of *C. thermophilum* Zuo1 with *S. cerevisiae*, *H. sapiens* and *B. taurus* homologs. Domains are marked with arrows above the sequence and colored accordingly. Specific kinks in the Zuo1 lever arm are indicated by gray arrows. Residues colored in green are important for two anchoring interactions of Zuo1 to the 80S ribosome.

We next determined the structures of RAC bound to 80S ribosomes using native *Chaetomium thermophilum* (*Ct*) complexes^25^ pulled out on Ssz1 (purified with endogenous Zuo1) for subsequent cryo-EM structure determination at 3.2 and 3.3 Å resolutions, with a local RAC resolution between 4.5 and 7 Å (Fig. 1, Extended Data Figs. 1 and 2 and Table 1). We obtained multiple 80S-RAC structures (with different ribosomal rotation states and two distinct conformations of RAC, denoted RAC-1 and RAC-2), with the extra-ribosomal factor RACK1 and the protective factor Stm1 bound as recently described for *Ct*80S ribosomes^26^. Although the ribosomes in the *Ct*80S-RAC complexes do not contain mRNA or tRNA, a mixture of NCs are visible from the peptidyl transferase center (PTC) to the very tunnel exit as described previously for *Ct*80S ribosomes^26^. The presence of NCs defines them as RNCs, as typically seen with translating ribosomes. The quality of the cryo-EM maps representing RAC allowed us to build complete models of this multidomain complex (Fig. 1a and Extended Data Fig. 2). Our recent X-ray structures of the RAC core, comprising Ssz1 with its SBD completed by the Zuo1 N terminus, could be readily placed as rigid bodies^6,8^. Although, for Zuo1-ZHD (zuotin homology domain) and the C-terminal four-helix bundle (4HB), structural models were available^20,22,27^, and large parts of Zuo1, including the J domain, the middle domain (MD) and linkers between the domains, had to be built de novo (Fig. 1b,c). Precise boundaries of the Zuo1 domains are shown in a multiple sequence alignment in Extended Data Fig. 3.

**Table 1:** Cryo-EM data collection, refinement and model statistics

	RAC-1 (EMD-14479) (PDB 7Z3N)	RAC-2 (EMD-14480) (PDB 7Z3O)
**Data collection and processing**		
Magnification	81,000	81,000
Voltage (kV)	300	300
Electron exposure (e^−^/Å^2^)	39.42	39.42
Defocus range (μm)	−0.8 to −2.5	−0.8 to −2.5
Pixel size (Å)	1.1	1.1
Symmetry imposed	*C*1	*C*1
Initial particle images (no.)	917,842	917,842
Final particle images (no.)	305,951	284,425
Map resolution (Å)	3.3	3.2
FSC threshold	0.143	0.143
Map resolution range (Å)	3.3–6.8	3.2–7.1
**Refinement**		
Initial model used (PDB code)	7OLC, 6SR6, 5DJE, 4GMQ, 2LWX	7OLC, 6SR6, 5DJE, 4GMQ, 2LWX
Model resolution (Å)	2.9	2.9
FSC threshold	0.143	0.143
Model resolution range (Å)	2.9–5.8	2.9–5.8
Map sharpening *B* factor (Å^2^)	–	–
Model composition		
Nonhydrogen atoms	213,544	213,453
Protein residues	12,785	12,787
Nucleotide residues	5,238	5,231
Ligands	ZN: 8	ZN: 8
	MG: 455	MG: 472
	ATP: 1	ATP: 1
*B* factors (Å^2^)		
Protein	225.32	185.30
Nucleotide	180.31	151.46
Ligand	130.84	121.47
R.m.s. deviations		
Bond lengths (Å)	0.003	0.003
Bond angles (°)	0.685	0.672
**Validation**		
MolProbity score	1.82	1.77
Clashscore	10.32	8.97
Poor rotamers (%)	0.12	0.10
Ramachandran plot		
Favored (%)	95.88	95.84
Allowed (%)	4.07	4.11
Disallowed (%)	0.05	0.06

**Extended Data Fig. 4:**
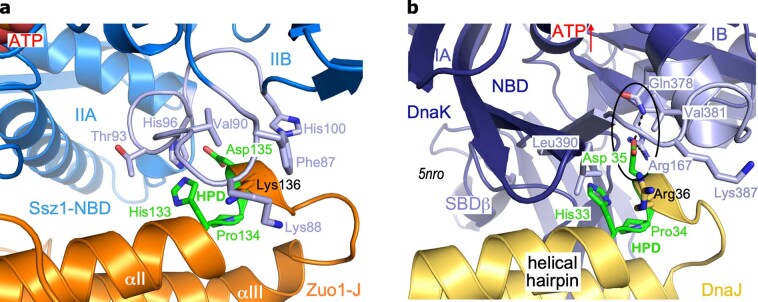
The HPD motif within the Hsp40/Hsp70 interaction. The conserved HPD motif within a helical hairpin of Hsp40 J domains forms the central element for the activation of ATP-hydrolysis in Hsp70 proteins. **a**, Zoom into the Ssz1-NBD/Zuo1-J interface of the RAC-1 complex (same in RAC-2). Ssz1 binds to but does not hydrolyze ATP. Residues involved in the interface around the HPD motif (green) are detailed. **b**, Zoom into the activating DnaK/DnaJ interface^1^, which is different and includes the linker of DnaK-NBD to its SBDβ. The central interaction network of the aspartate within the HPD motif is encircled. The position of ATP is indicated by an arrow.

In our RAC structures, Zuo1 contacts the Ssz1-SBD mainly by the previously described tight interaction with Zuo1N (residues 1–72; 3,060-Å^2^ interface area with a Δ*G* of −42.4 kcal mol^−1^, 80% of the total Ssz1-Zuo1 interface)^6,8^. A conserved polyproline type-II helix (LP-motif) at the Zuo1 N terminus binds to the Ssz1-SBD as a pseudo-substrate^6,8^. The Ssz1-Zuo1N interface is now enlarged by an extension of Zuo1N-αI and an additional α-helix (Zuo1N-αII, residues 62–72; Fig. 1b and Extended Data Fig. 3) that entangle the Ssz1 specific linker and its helix (αL) connecting NBD and SBD. A linker between Zuo1N and the J domain (residues 73–87) is highly negatively charged and barely contacts the Ssz1-SBD and Zuo1 J domain (residues 88–175). The J domain shows the canonical fold of JDPs, with four α-helices, including a central helical hairpin^28^ (helices J-αII and J-αIII) that forms the only contact between the J domain and Ssz1-NBD (685 Å^2^, Δ*G* of −2.7 kcal mol^−1^). This hairpin bridges lobe IIA to IIB and contains the conserved HPD motif (*Ct*Zuo1 His133-Pro134-Asp135), which is crucial for Hsp70 activation^28^. The HPD motif breaks helix J-αII at its C terminus and is completely masked by its Ssz1-NBD interaction (Extended Data Fig. 4a). This rather unspecific contact differs from the classical Hsp70/JDP activating complex^29^, where the HPD motif interacts with the conserved Hsp70 linker region, inserts the helical hairpin between NBD lobes IA and IB, and also contacts SBD-β (Extended Data Fig. 4b). Of note, this canonical contact is also small and unstable (925 Å^2^, Δ*G* of −1.3 kcal mol^−1^), which is reflected by a generally transient Hsp70/JDP interaction^7^.

In contrast to the Zuo1 N-J connection, the Zuo1-ZHD (residues 176–289) is directly linked to the J domain, which together form a rigid entity (Fig. 1a–c). The ZHD closely corresponds to an X-ray structure for yeast Zuo1-ZHD (root-mean-squared deviation (r.m.s.d.) of 2.0 Å)^22^ and comprises a three-helix bundle with an extended C-terminal α-helix (ZHD-αIII) (Fig. 1c). The ZHD was previously characterized as a ribosome-binding domain^22^, but its interactions within RAC were not resolved. Our structures reveal an intimate Zuo1-ZHD contact with Zuo1-J, mostly to J-αIV, flanked by loop interactions involving salt bridges and stacking of aromatic residues (buried surface area of 623 Å^2^, Δ*G* of −5,2 kcal mol^−1^; Fig. 1c and explanations therein). We also observe an additional small contact between Zuo1-ZHD and Ssz1-NBD, which involves two aspartates adjacent to ZHD-αIII (Fig. 1c and explanations therein). This contact changes between the two distinct conformations of RAC on the 80S ribosome (RAC-1 and RAC-2). Between the ZHD and the following helical MD (residues 290–354) we observe a *π*-cation stacking network fixing the first two turns of MD-αI to the ZHD (Fig. 1c). The MD connects the three N-terminal Zuo1 domains to the rigid C-terminal four-helix bundle (4HB; residues 355–446), which anchors RAC on the 40S subunit by interacting with the rRNA expansion segment ES12^20^. Taken together, our data allow us to build a complete model of RAC with precisely defined domain boundaries and to describe interactions within Zuo1 as well as with Ssz1, which differ from canonical Hsp70/JDP interactions.

### Two distinct conformations of RAC on the 80S ribosome

**Fig. 2:**
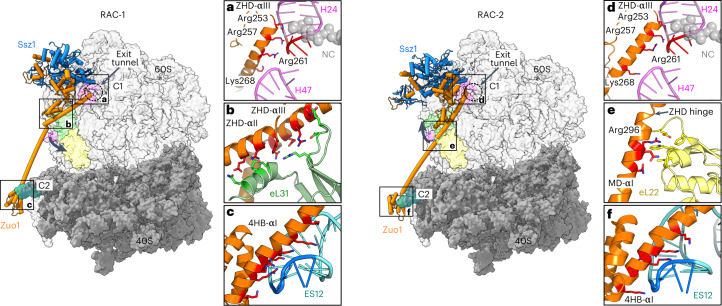
RAC interactions with the 80S ribosome. Cryo-EM structures of *Ct*RAC bound to the 80S ribosome in two distinct conformations: RAC-1 (left) and RAC-2 (right). The main 80S contacts are highlighted with squares that correspond to the zoomed images in **a**–**f**. **a**,**d**, ZHD-80S interaction (C1 contact) with H24 and H47 of the 26S rRNA in RAC-1 (**a**) and RAC-2 (**d**). C1 is formed by the N-terminal end of the lever arm (ZHD-αIII) at the rim of the ribosomal tunnel exit. **b**, ZHD interaction with the eL31 ribosomal protein in RAC-1. **e**, ZHD-MD interaction with the eL22 ribosomal protein in RAC-2. **c**,**f**, 4HB interaction (C2 contact) with ES12 of the 18S rRNA in RAC-1 (**c**) and RAC-2 (**f**). C2 is formed at the C-terminal end of the lever arm (Zuo1-4HB) and the closing tetraloop (1695-GCAA, highlighted in blue) of ES12 in the 40S subunit.

**Extended Data Fig. 5:**
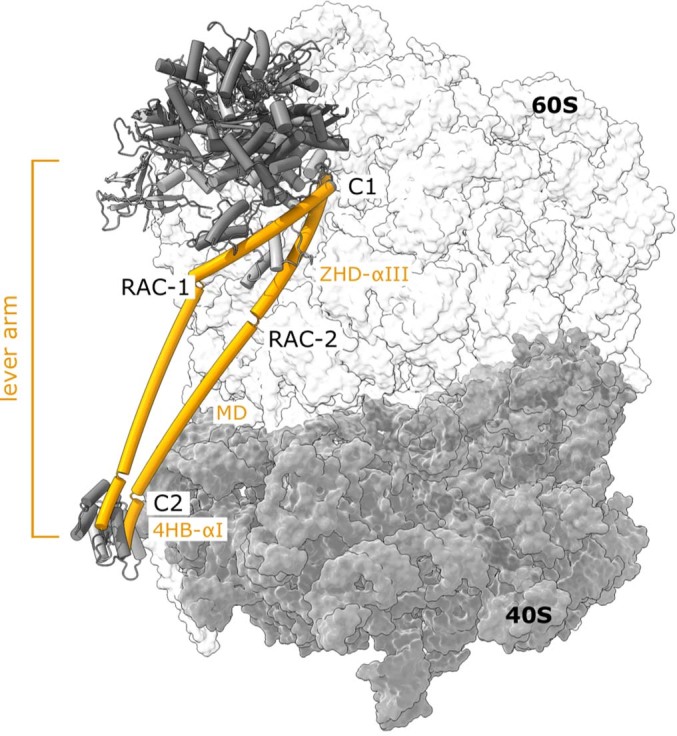
Two RAC conformations on the 80S ribosome. RAC adopts two discrete conformations (RAC-1 and RAC-2) on the 80S ribosome, shown here in superposition. Interactions with the ribosome are formed through multiple contacts of a Zuo1 lever arm (shown in orange cartoon) that includes the helix αIII of the ZHD, the entire MD, and the helix αI of the 4HB. Ribosome is shown in surface representation. Two main contacts affixing the Zuo1 lever arm at the ribosome are labeled C1 and C2.

**Extended Data Fig. 6:**
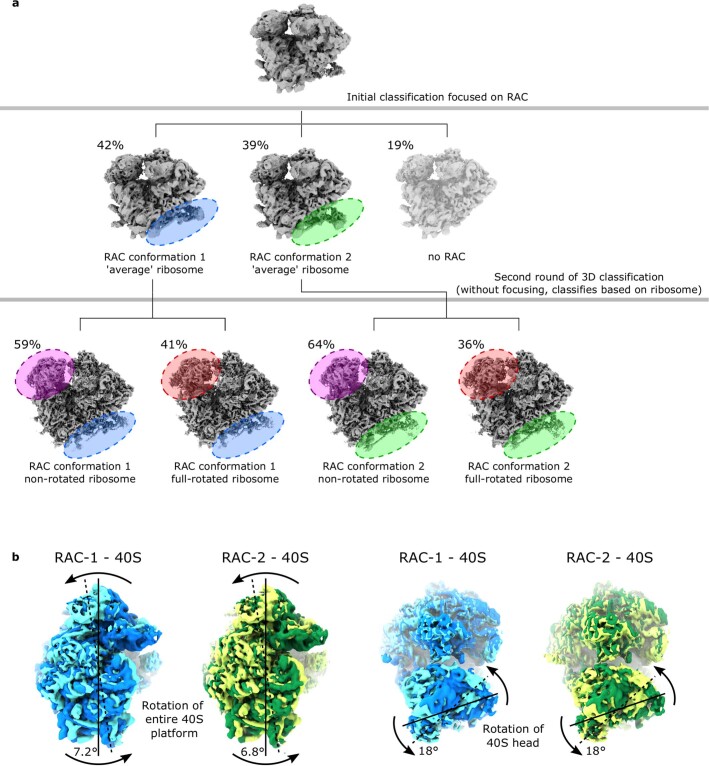
Extended processing of the cryo-EM data and 3D variability analysis. **a**, Two 3D classes containing RAC (see Extended Data Fig. 1, here marked with blue and green circles) were further analyzed by a second round of 3D classification focused on the ribosome 40S head (marked with red and magenta circles). Such classification allowed to separate fully rotated 80S ribosomes (red) from non-rotated 80S particles (pink) (40S head is main indication in the rotation movement). Classification showed that both RAC conformations (RAC-1 and RAC-2) can accommodate full ribosomal rotation, and contain similar percentage (59% RAC-1 vs. 64% RAC-2 and 41% RAC-1 vs. 36% RAC-2) of non-rotated and full-rotated 80S ribosomes. **b**, 3D variability analysis of ribosomal rotation. RAC-1 is shown in blue colors, and RAC-2 is shown in green. Overlaid maps (low-pass filtered) are the first frame and the last frame of the 3D variability analysis. Left panels show the rotation of the 40S body (about 7°), and right panels visualize the rotations (swiveling) of the 40S head (when body is aligned, 18°). Rotations were measured in UCSF-Chimera using the command ‘measure rotation‘.

Consistent with previous data^3,6,20,21^, our RAC-80S complexes display an extended RAC structure that spans more than 200 Å and contacts both ribosomal subunits. RAC adopts two distinct conformations (RAC-1 and RAC-2) on a rotating ribosome (Fig. 2, main panels and Extended Data Fig. 5). This ratchet-like motion is a conserved feature of all ribosomes and is intrinsic to mRNA/tRNA translocation^30^. Three-dimensional (3D) variability analysis^31^ allowed us to visualize continuous movement of the 40S subunit with respect to 60S for both RAC conformations (Extended Data Fig. 6a and Supplementary Video 1). It was previously thought that RAC stabilizes the 80S ribosome in the non-rotated state and that its movement is coupled to ribosomal rotation^21^. However, our structures demonstrate that idle 80S ribosomes containing RAC in either conformation exhibit the same distribution of rotational states (Extended Data Fig. 6b and Supplementary Video 1). Rotation of the entire 40S body, except the ES12 movements, in both cases reaches ~7°, and swiveling of the 40S head reaches up to 18°. For better comparison, RAC-1 and RAC-2 were built on the non-rotated ribosome.

In both RAC conformations, interactions with the ribosome are exclusively formed through Zuo1 by a lever arm (residues 253–371; Extended Data Fig. 5) that we define based on our structures to include ZHD-αIII (residues 253–289), the entire MD (residues 290–354) and 4HB-αI (residues 355–371) (Fig. 2, main panels). Ssz1 does not interact with the ribosome but is kept in close proximity to the ribosomal tunnel exit by its interaction with Zuo1N^6,8^ and by the two small contacts between Ssz1-NBD with the Zuo1 J-ZHD unit.

Although a previous study suggested that the RAC-ribosome interaction changes with ribosomal rotation^21^, our data clearly show that the Zuo1 lever arm anchors RAC at the ribosome with two main contacts (C1 and C2) that are maintained in both RAC conformations independent of the ribosomal rotation state. C1 is formed by the N-terminal end of the lever arm at the rim of the ribosomal tunnel exit (Fig. 2a,d) with three conserved arginines from ZHD-αIII (Arg253, 257 and 261; for homology see Extended Data Fig. 3). These arginines form a so-called arginine-rich motif (ARM)^32^ that affixes Zuo1 in the major groove of the tetranucleotide loop (tetraloop, 376-GAAA) at the tip of helix H24 of 26S rRNA. The interaction is completed by the positive N-terminal helix dipole of ZHD-αIII, which positions the helix on the phosphoribose backbone. In yeast, the corresponding arginines 247 and 251 also contact H24 of the 26S rRNA^22^, and disruption of this contact completely abolishes RAC binding to the ribosome in yeast, both in vitro and in vivo^33^.

C2 is formed at the C-terminal end of the lever arm between Zuo1-4HB and the closing tetraloop (1695-GCAA) of 18S rRNA ES12 in the 40S subunit (Fig. 2c,f). Similar to C1, C2 also involves an elaborate ARM interaction between 4HB-αI and ES12. The helix contributes two arginines (Arg362 and 365) and five lysines (Lys350, 354, 358, 359 and 369) to this interaction. Although ES12 shortening severely affected translation fidelity and the readthrough effects of stop codons, the RAC-ribosome interaction was only mildly destabilized^22^.

**Extended Data Fig. 7:**
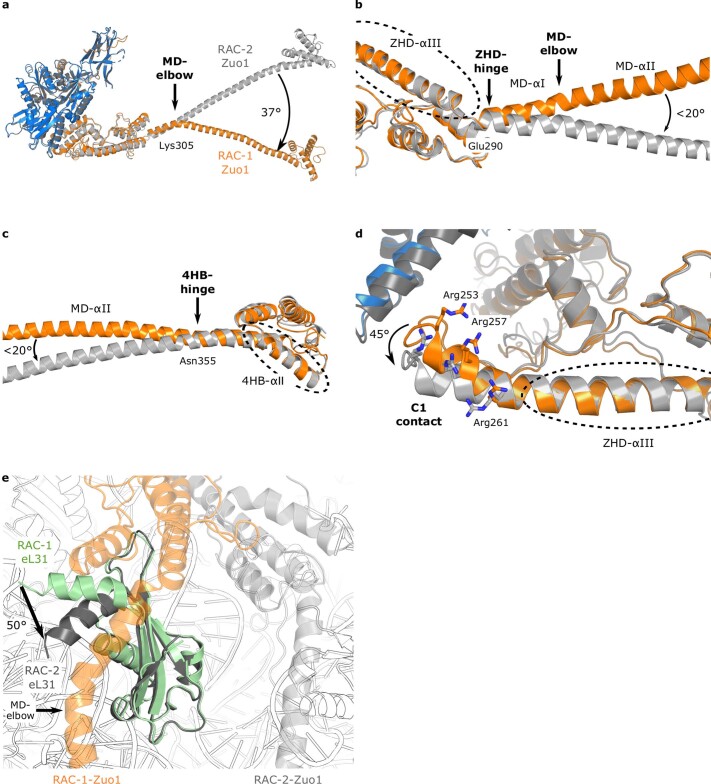
Rearrangements of the Zuo1 helical lever arm and ZHD anchor region in different RAC conformations. **a**, Full view of the *C. thermophilum* RAC, when superposed on Ssz1. RAC-1 corresponds to a bent MD-elbow (orange) and RAC-2 to a straightened arm (gray). **b-c**, Zoom in views of ZHD-hinge, MD-elbow and 4HB-hinge. **d**, Rearrangements in the Zuo1-ZHD anchor region (45° rotation). In **b** to **d** superposition is performed on the areas marked by dashed circle**. e**, Changes in eL31 N-terminal helix in different RAC conformations. The N-terminal helix of eL31 is rotated by 50° in RAC-1 in respect to RAC-2. RAC-1 eL31 is shown in green cartoon, RAC-2 eL31 is given in gray. The ribosome is depicted as a transparent cartoon, with Zuo1 shown in orange.

The C1 and C2 contacts appear invariant in both RAC conformations. However, the lever arm undergoes a complex motion, which can be described by a bending elbow located in the MD (here denoted as the MD-elbow at Lys305; Extended Data Fig. 7a). Although, in RAC-1, the MD-elbow is bent by 37°, it is straightened in RAC-2 (Fig. 2, main panels). In addition, two minor hinges (<20°) localize at both ends of the lever arm, between ZHD and MD (ZHD hinge at Glu290) and between MD and 4HB (4HB-hinge at Asn355) (Extended Data Fig. 7b,c). Interestingly, when RAC-1 and RAC-2 are superposed on Ssz1 (Extended Data Fig. 7), Ssz1 and Zuo1 J-ZHD (as well as the 4HB by itself) overall behave as rigid bodies (r.m.s.d. of <1.3 Å). However, as both ends of Zuo1 are fixed on the ribosome, the invariant C1 and C2 contacts must somehow accommodate changes within the MD-elbow. Indeed, when comparing the RAC-1 and RAC-2 contacts with the ribosome, the Zuo1-ZHD rotates around C1 (residues 246–261) with respect to the J-ZHD unit (45° rotation at borders) (Extended Data Fig. 7d), and C2 is maintained by a substantial bending of ES12 (Fig. 2c,f).

**Extended Data Fig. 8:**
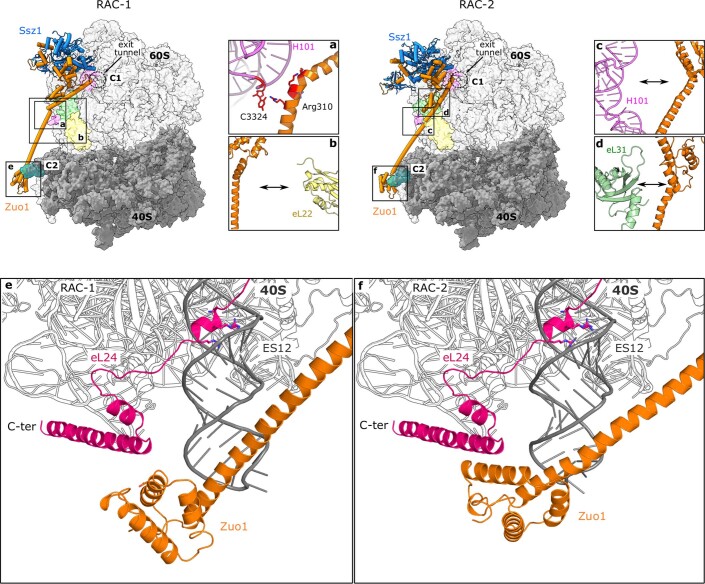
RAC interactions with the 80S ribosome. The main interaction sites of RAC-1 and RAC-2 (shown in the main panels) are highlighted with squares that correspond to the zoom images **a** to **d**. **a, c**, Zuo1 MD-αI interaction with H101 of 26S rRNA in RAC-1 (**a**) and its abolishment in RAC-2 (**c**). **b**, Zuo1-ZHD domain is shifted away from eL22 in RAC-1 (compare with Fig. 2e for C2). **d**, ZHD-MD domains shifted away from eL31 in RAC-2 (compare with Fig. 2b for RAC-1). **e-f**, A ribosome-internal arginine-rich motif (ARM) is provided by eL24 of the 60S subunit and holds ES12 in a fixed location by binding in a widened major groove in both RAC-1 (**e**) and RAC-2 (**f**) conformations. The C-terminal helix of the eL24 anchors on the 40S body of the ribosome and is positioned close to the Zuo1-4HB domain. **e-f** zoom images are rotated for better representation.

Apart from C1 and C2, there are several interactions between the lever arm and the ribosome that are adjusted. In RAC-1, Zuo1-ZHD interacts with protein eL31 via a mixed polar–apolar helical bundle (ZHD-αII and eL31 N-terminal helix) and multiple salt bridges between the lever arm (ZHD-αIII) and an internal eL31 loop (Fig. 2b). This interaction nicely correlates with previously observed crosslink data^22^. Interestingly, the eL31 N-terminal helix is rotated by 50° towards the ZHD compared to RAC-2 (and the idle 80S ribosome^26^; Extended Data Fig. 7e). Furthermore, in RAC-1 the MD-elbow rests on the 26S rRNA 3′-end (H101), with Arg310 seemingly stacking on a bulged-out cytosine (C3324) (Extended Data Fig. 8a).

**Fig. 3:**
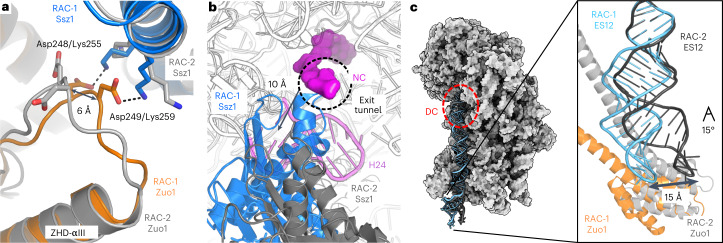
Details of the structural differences between RAC-1 and RAC-2. RAC-1 is shown in color (Ssz1, blue; Zuo1, orange) and RAC-2 in gray. **a**,**b**, Contact between Zuo1-ZHD and Ssz1-NBD close to the tunnel exit. In RAC-1, this contact enables two salt bridges (Zuo1-Asp248/Ssz1-Lys255, Asp249/Lys259), which are not possible in RAC-2 (shift of 6 Å, **a**). The contact between Zuo1 and Ssz1 is broken, and Ssz1-NBD moves 10 Å away from the tunnel exit. (**b**). NC is shown in magenta and represented as surface. 26S rRNA H24, which is involved in the C1 contact with Zuo1, is shown in pink. **c**, The 40S-Zuo1 contact. Left: 40S is shown in surface representation (gray), with ES12 of the 18S rRNA shown in sticks, and the decoding center (DC) highlighted by a red circle. Right: zoom-in view of the Zuo1-4HB interaction (C2 contact) with ES12 in both RAC conformations. The 4HB-ES12 contact stays invariant, but the tip of ES12 adapts by a 15° bend and moves by 15 Å.

In RAC-2, these interactions have disappeared (Extended Data Fig. 8c,d), and straightening the MD-elbow moved the lever arm by up to 40 Å on top of protein eL22, which fixes the ZHD hinge by two internal loops and its very C terminus (Fig. 2e). In particular, Zuo1 Arg296 is involved in *π*-cation stacking with a tryptophan and in a salt bridge. Previous crosslinking studies failed to detect the eL22 contact, probably due to technical reasons^22^. Finally, adjacent to C1, a weak contact between H47 and a single lysine (Lys268) is observed in RAC-2, which is lost in RAC-1 (Fig. 2a,d). Another striking difference is observed next to the tunnel exit at the contact between Zuo1-ZHD and Ssz1-NBD (Fig. 3a,b). In RAC-1, this contact enables two salt bridges of juxtaposed residues (Zuo1-Asp248/Ssz1-Lys255, Asp249/Lys259), which, due to an increased distance, cannot be formed in RAC-2, resulting in Ssz1-NBD moving 10 Å away from the tunnel exit.

Although at C2 the contact with ES12 stays invariant in both RAC conformations and throughout ribosomal rotation, the tip of ES12 adapts by a 15° bend in RAC-2, independent of 40S body rotation (Fig. 3c). The tip of ES12 thus moves by 15 Å. Interestingly, next to its flexible tip, ES12 is held in place by another ribosome-internal ARM, this time provided by eL24 of the 60S subunit, which is threaded through a widened ES12 major groove and with its long C-terminal helix anchored on the 40S body (Extended Data Fig. 8e,f). Furthermore, ES12 forms the end of the long 18S rRNA helix H44 located between the 40S and 60S subunits (length, 200 Å) that reaches up to the codon-anticodon base pairs and contacts Stm1, which occupies the P-site, as described recently^26^. H44 is known to ensure the accuracy of translation elongation and termination^22^, but further investigation is needed to delineate the exact role of RAC in translational fidelity. Overall, we observe RAC in two distinct conformations on a rotating ribosome and resolve mechanistic details of RAC-80S interactions (Supplementary Video 1).

### Model of Ssb stimulation by Zuo1

**Extended Data Fig. 9:**
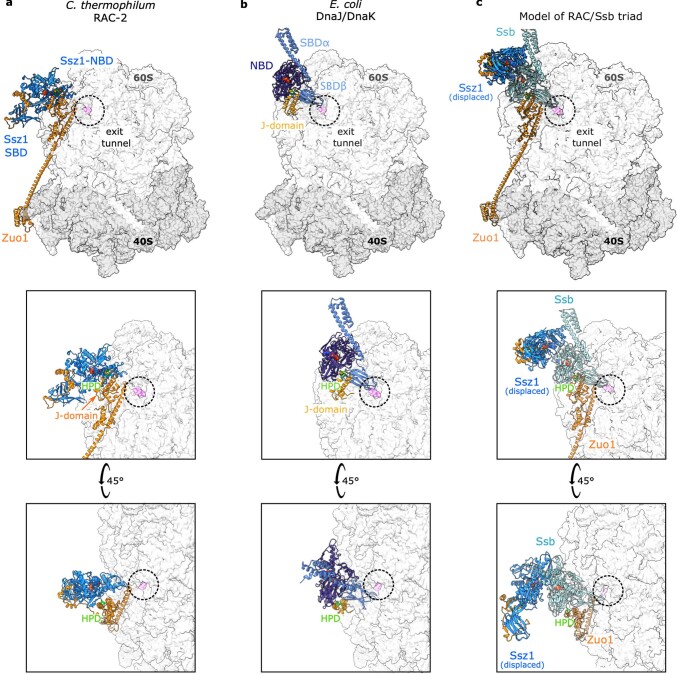
Model of the RAC/Ssb triad on the ribosome. **a**, RAC-80S complex in RAC-2 conformation. The 80S ribosome is shown in a transparent surface representation (60S: pale gray, 40S: dark gray, NC: magenta), Zuo1 is shown as orange cartoon, and Ssz1 as blue cartoon. **b**, The *E. coli* DnaK/DnaJ complex^29^, with the J domain of DnaJ superposed on the Zuo1 J domain of the RAC-2 complex. **c**, DnaK (from panel **b**) is replaced by yeast Ssb (open, ATP-bound state)^34^ to obtain a model for Ssb activation by Zuo1-HPD. In this model, the Ssz1-NBD that masks the Zuo1-HPD motif needs to detach from the Zuo1 J-ZHD at the ribosomal tunnel exit. Thus, Ssz1-NBD is moved to a position matching a crystallographic Ssb homodimer^34^ (PDB code 5tky), in agreement with recent Ssz1/Ssb cross-linking studies^24^. Ssz1-SBD bound to Zuo1-N is kept in close neighborhood to the ribosome-docked parts of Zuo1 by the short Zuo1 N-J linker.

RAC forms a functional chaperone triad with Ssb, which needs activation by Zuo1-J for productive interaction with NCs. Our structures of RAC at the 80S, and structures of the *Escherichia coli* DnaK/DnaJ complex^29^ and of yeast Ssb (open, ATP-bound state)^34^ allow us to derive a structure-based model of the RAC/Ssb triad at the ribosome. First, the DnaJ J domain is superposed on Zuo1-J (RAC-2 chosen, RAC-1 also possible). Second, DnaK (in the DnaK/J complex) is replaced by Ssb^34^ to obtain a model for Ssb activation by the Zuo1 HPD motif (Extended Data Fig. 9). In the superposition of the J domains, the NBDs of DnaK and Ssz1 would clash. The Ssz1-NBD that masks the Zuo1 HPD motif (described above) needs to detach from the Zuo1 J-ZHD unit, which is anchored at the ribosomal tunnel exit by C1. Noteworthy, in RAC-2 the slight detachment of Ssz1-NBD from Zuo1-ZHD (moved away from the tunnel exit by 10 Å compared with RAC-1) already opens this weak contact and provides access to the tunnel exit. The short Zuo1 N-J linker (15 residues) will keep Ssz1-Zuo1N in close neighborhood. Superimposing Ssb on DnaK places Ssb-SBD-β directly on top of the tunnel exit ready for interaction with short NCs, consistent with previous crosslink and ribosome profiling data (Extended Data Fig. 9c)^3,17^. In the ATP-bound open state, the Ssb-SBD-α lid domain is not interfering with any contacts and points away from the ribosome. This seems counterintuitive, as the lid domain harbors the key ribosome-binding motif of Ssb. However, the structures of Ssb-ATP and DnaK-ATP have been obtained by fixing the domain arrangement by an engineered disulfide bridge^34,35^. In addition, autonomous ribosome-binding of Ssb is not required for its function in the presence of RAC^36^.

**Extended Data Fig. 10:**
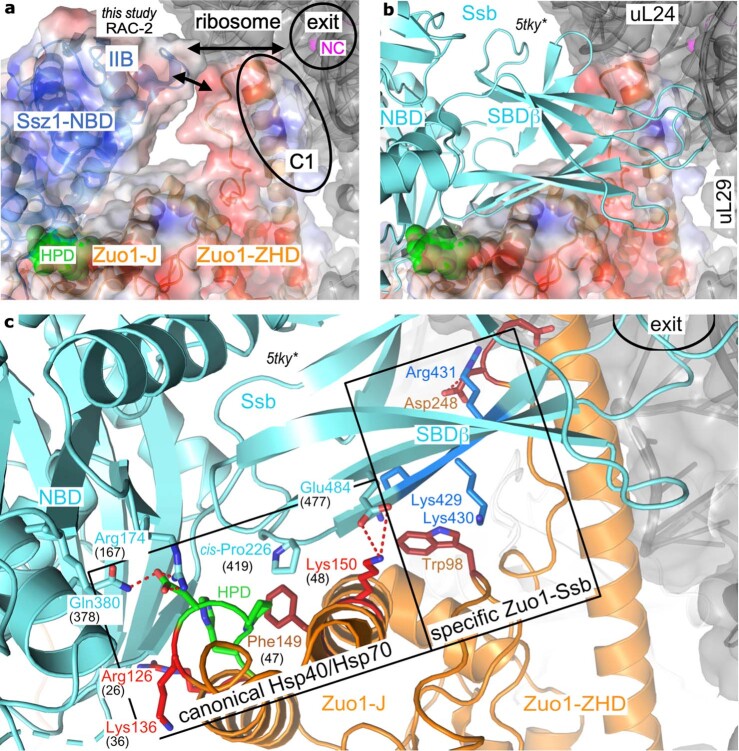
Model for the activated RAC-Ssb complex. **a**, Zoom on the RAC-2 structure at the major C1 contact of Zuo1-ZHD next to the ribosomal tunnel exit. Surfaces are colored for RAC according their surface potentials (±5 kbTe; blue: positive, red: negative). The HPD motif is highlighted in green. In RAC-2, Ssz1-NBD is displaced from the tunnel exit and the contact to Zuo1-ZHD is broken (indicated by arrows). **b**, Same view with Ssb-ATP from *C. thermophilum* (cyan) bound to the HPD motif in the activating position (modeled as described in main text). Ssb-SBDβ locates next to negatively charged surface patches of Zuo1 ready to accept substrate NCs, in line with cross-links between uL24 and uL29 with Ssb-SBDb^24^. **c**, Zuo1-Ssb interactions extend the canonical Hsp40/Hsp70 contact around the HPD motif by specific interactions of Zuo1-ZHD with Ssb-SBDβ. The interaction in our model involves a conserved basic motif in Ssb-SBDβ (here: KKR 429-431, highlighted in blue) that has previously been assigned to ribosome binding^36^. Interactions are detailed for the *C. thermophilum* complex (residue numbers in parenthesis correspond to the DnaK-DnaJ complex of *E. coli*^29^).

In contrast to most Hsp70 chaperones that can be activated by several JDPs, it has been shown that Zuo1 is the only JDP that activates Ssb and stimulates ATP hydrolysis^5^. However, the basis of this specificity is not clear. Our model with Ssb in the activating position does not show any clashes with Zuo1 or the ribosome, and the Ssb-Zuo1-J interface shows all characteristic interactions described for the DnaK–DnaJ complex^29^ (Extended Data Fig. 10). In addition to these canonical Hsp70/JDP interactions, our model also visualizes Ssb-specific interactions with Zuo1. Interestingly, these specific interactions mainly involve a KKR-motif (residues 429–431; KRR-motif in *Sc*Ssb) in Ssb-SBD-β that has previously been described as a ribosome attachment point^24,36^. In our model, however, the two lysines embrace Zuo1-J Trp98, while the arginine forms a salt bridge with Zuo1-ZHD Asp248 (Extended Data Fig. 10c) that replaces the interaction with Ssz1-NBD observed in RAC-1 (but not in RAC-2). Therefore, our structure-based model suggests that the KKR-motif contributes to the specific activation of Ssb by Zuo1.

## Discussion

**Fig. 4:**
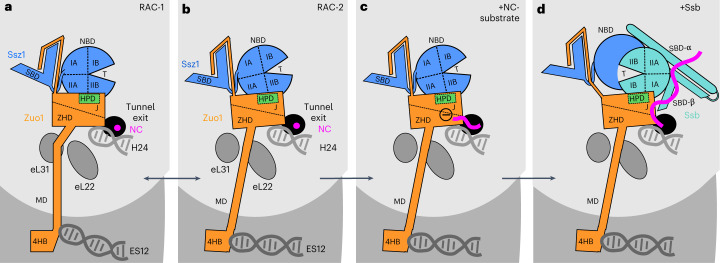
Structure-based model for RAC/Ssb action at the 80S. **a**–**d**, Integrating our cryo-EM structures with the current data on RAC and Ssb allows us to devise a detailed model of RAC/Ssb action at the ribosome. RAC binds to the 80S in two distinct conformations, with Zuo1 possibly oscillating between RAC-1 and RAC-2 (**a**,**b**). The HPD motif of Zuo1-J (green) is masked by Ssz1-NBD. The RAC/Ssb substrate (positively charged NC) emerging from the exit tunnel first interacts with a negatively charged patch in Zuo1 (**c**). Elongation of the NC allows it to reach a positively charged patch in Ssz1 (not indicated). The NC pushes the Ssz1-NDB away and thereby frees the HPD motif. This allows the Zuo1-Ssb interaction, and the growing NC contacts Ssb, which can now be stimulated by Zuo1-J (**d**). Ssb is positioned next to the tunnel exit, with its SBD conveniently placed close to the emerging NC, and with its NBD forming a heterodimer with Ssz1-NBD. When the Ssz1-NBD is displaced from the HPD by NC and Ssb binding, the Ssz1-SBD stays tied up with Zuo1-N. After stimulation of ATP hydrolysis, Ssb can detach from the ribosome, and Ssz1-NBD returns to shield the HPD motif.

The RAC-80S structures described here provide details of the RAC architecture and RAC/80S interaction. The contacts observed between 80S ribosomes and RAC localize this specific Hsp40/Hsp70 activity at the ribosomal tunnel exit and provide an answer to the function of Ssz1 and the specificity of the Zuo1/Ssb pair. Together with previously obtained crystal structures of JDP/Hsp70 complexes^29,37^ and Ssb^34^, the RAC-80S structures allow us to extend our RAC/Ssb model and propose a mechanism for the action of the RAC-Ssb chaperone triad on the ribosome (Fig. 4). The mechanism is based on our observation that the strong ARM contacts of Zuo1 stay invariant during ribosomal rotation and that the ZHD/J domain entity behaves as a rigid body. Thus, it can be assumed that RAC remains attached to the RNC complex during protein biosynthesis and the J domain position adapts to the observed RAC conformations. The second premise is that the activating JDP/Hsp70 interaction, mediated by the HPD motif, is universally conserved and that the available crystal structures can serve as a general template. However, in the non-activating case of Zuo1/Ssz1, the HPD motif is completely masked by its Ssz1-NBD interaction. Furthermore, NC binding contributes to RAC/Ssb interaction at the ribosome, and specific sequence requirements for the Ssb/NC interaction have been determined by ribosome profiling^17^. Ssb binds to degenerated sequence motifs enriched in positively charged and hydrophobic residues positioned at a distance of 35–53 residues from the PTC^17^, and crosslinking data indicate that the NC is handed over in a relay from Zuo1 via Ssz1 to Ssb^8^. In the absence of functional RAC, Ssb fails to interact with NCs, as the high-affinity substrate-binding state of Ssb is not induced^15,34^. Our structures now localize the Zuo1-ZHD next to the tunnel exit and show that it not only modulates ribosome and Ssz1 interactions, but also exposes a highly negatively charged surface in a matching distance from the PTC. The adjacent Ssz1-NBD IIB lobe is also negatively charged (and slightly hydrophobic), while the more distal interface to the IA lobe is strongly positively charged. Our current model integrates these observations, and suggests that complementary charges might contribute to NC binding and handover. Positively charged NCs first interact with Zuo1-ZHD, whereas slightly longer NCs can bind to adjacent negative and slightly hydrophobic patches in Ssz1-NBD lobe IIB (Extended Data Fig. 10a). Further elongation of the NC and the dynamic Zuo1-ZHD/Ssz1-NBD contact, as observed between the RAC-1 and RAC-2 complexes, can then direct the NC towards the positive patch between Ssz1-NBD lobes IB and IIB, and are probably sufficient to dissociate the weak contact between Ssz1-NBD and the Zuo1 HPD motif. This would allow Ssb to join and engage in the canonical activating, but transient J domain contact (Extended Data Fig. 10b). Shielding of the HPD motif by Ssz1 avoids premature activation of Ssb at the ribosome, so activation of ATP hydrolysis in Ssb is coupled to the NC interaction before Ssb (in the adenosine diphosphate state) dislodges from the ribosomal surface. The J domain can then again be masked by Ssz1 to avoid unproductive engagements, for example, with another Ssb molecule.

The position of Ssb at the ribosome has remained quite puzzling, despite several crosslink studies^3,5,19–24^. Recent data place Ssb next to the tunnel exit with different binding modes (with bound ATP or adenosine diphosphate)^24^. Furthermore, these data specify interactions between Ssz1-NBD with both Ssb-NBD and Ssb-SBD-α and suggest the formation of an Ssz1-Ssb NBD heterodimer. This placement of Ssb nicely correlates with our cryo-EM structures and supports our structure-based model (Fig. 4 and Extended Data Fig. 10c). Notably, the proposed heterodimer interaction resembles homodimers observed in crystals of the Hsp70s Ssb^34^ and DnaK^38^, and also the Hsp110 Sse1^39^, suggesting that NBD dimer formation might be more common in Hsp70 and Hsp110 chaperones.

The two distinct RAC conformations and their ribosome contacts observed in this study do not correlate with ribosomal rotation, and RAC is predicted to be very flexible when in solution^20^. Therefore, a question remains as to what triggers RAC-1/RAC-2 oscillation, and how the entire chaperone triad is coupled to translation on the one hand and to Ssb binding and ATP hydrolysis on the other. It is tempting to speculate that factors missing in our study might be involved, for example, the complete mRNA•tRNA_2_ module, Ssb and a steadily growing NC that harbors Ssb-substrate sequences.

In this respect, our data are complemented by a parallel cryo-EM study describing different RAC-80S complexes from bakers’ yeast at similar resolution^40^. Briefly, that paper describes endogenous 80S/RAC, RNC/RAC substituted with recombinant RAC and finally RNC/RAC/Ssb. In contrast to the current study, the yeast structures report on N- and C-terminal parts of RAC but not on complete RAC. Some of these structures contain tRNA, a NC and Ssb. However, irrespective of the presence of these factors, the authors also see no correlation of RAC binding with ribosome rotation, and contacts of Zuo1 with the ribosome do not change. This suggests that the presence of tRNA does not determine the RAC-ribosome interaction. In the presence of a defined NC substrate, Ssz1 is dynamic and observed in a different orientation with respect to Zuo1^40^. This allows Ssz1-SBD to approach the ribosomal tunnel exit as anticipated from crosslink studies^8^, and together with our data, this visualizes the dynamic nature of the Ssz1/Zuo1-J interaction. Ssz1 masks the Zuo1-J HPD motif in different ways involving the Ssz1 NBD or the SBD completed by Zuo1-N. Finally, the presence of Ssb induces structural changes resulting in a loss of information for Ssz1^40^, which seems displaced from the HPD motif. In this structure, Ssb is also not visible, as probably expected from the typically transient interaction between JDPs with their cognate Hsp70 proteins, and the whole chaperone triad appears less defined. Although a detailed comparison of the yeast^40^ and our *C. thermophilum* structures is not possible as the yeast coordinates are not deposited, the interactions described in the yeast study largely agree with our study.

What about the influence of RAC on the fidelity of translation^22,41^? Here, ES12 plasticity within the 40S subunit might play an important role. The yeast structure for RNC-RAC-Ssb^40^ nicely correlates with our data for the impact of the 4HB interaction on H44. Specifically, the local adjustment at the tip of H44 including ES12 is conserved in yeast and *C. thermophilum*. Previous studies have already investigated the Zuo1-4HB interaction with ES12^22,41^, but so far only with perturbed or truncated systems. It was previously envisaged that a direct coupling between RAC binding and the decoding center occurs via the central rRNA helix H44 including ES12 at its tip^23^. The structural data suggest that the influence of RAC on fidelity might depend on modulating the speed of ratcheting, for example, when switching between RAC-1 and RAC-2. Further functional and especially high-resolution structural studies of all components of stalled on-pathway complexes are needed to eventually unveil the complete video of this unique cotranslational chaperone triad in protein biosynthesis. The absence of a Ssz1 homolog in humans and the presence of additional domains in *hs*Zuo1 together with off-ribosomal transcriptional functions of Zuo1 and Ssz1^23,42^ promise further surprises from this puzzling Hsp70 chaperone system.

## Methods

### Construct design, cloning and expression

pRSFduet-ctSSZ-FTpA was used for ectopic integration and expression of SSZ-FTpA in *C. thermophilum*. The SSZ promoter region (628 bases) and open reading frame were amplified by polymerase chain reaction from *C. thermophilum* genomic DNA (primers: SszF_EcoRI: GGAATTCGATGGCGCGCTGGTTGTG and SszR_BamHI: CGGGATCCCTACGCGCTCAGCGTGCCG) and fused to the Flag-TEV-protA tag, resulting in the pRSFduet-ctSSZ-FTpA plasmid. *C. thermophilum* wild-type strain was transformed with the pRSFduet-ctSSZ-FTpA plasmid as described in ref. ^25^. In brief, protoplasts were generated from the cell wall digestion of the fungus mycelium and mixed with the linearized plasmid DNA. The transformed protoplasts were plated and selected on complete culture medium^25^ with sorbitol agar plates, supplemented with 0.5 mg ml^−1^ terbinafine, incubated at 50 °C for 3 days. Expression of the SSZ-FTpA protein was verified by western blotting of whole-cell lysate using PAP (Sigma-Aldrich, P1291) antibodies according to the manufacturer´s protocol (1:2,500 dilution).

ctSSZ1-FTpA mycelia were cultivated in a rotary shaker at 55 °C, collected through a metal sieve, washed with water, dried with a vacuum filter and immediately frozen in liquid nitrogen. Frozen mycelium cells were ground to fine powder by CryoMill (Retch; 5 min, frequency 30 s^−1^) and stored at −80 °C.

### Purification of *C. thermophilum* 80S-RAC complexes

The powdered mycelium was resuspended in 20 mM HEPES-KOH (pH 8.0), 150 mM NaCl, 50 mM KOAc, 2 mM Mg(OAc)_2_, 1 mM DTT, 5% glycerol and 0.1% NP-40. Insoluble material was removed by centrifugation (35,000*g*, JA25-50 rotor (Beckman), 30 min). The lysate was transferred onto immunoglobulin-G beads and incubated at 4 °C for 15 h. Beads were washed (20 mM HEPES-KOH (pH 8.0), 150 mM NaCl, 50 mM KOAc, 2 mM Mg(OAc)_2_, 1 mM DTT, 5% glycerol, 0.01% NP-40), incubated with tobacco etch virus (TEV) protease at 4 °C for 4 h and eluted. The elution fractions were pooled together and precipitated by adding 7% wt/vol of PEG20000. After a 10-min centrifugation, the pellets were resuspended in 20 mM HEPES-KOH (pH 7.5), 50 mM KOAc, 5 mM Mg(OAc)_2_, and 2 mM DTT and used for cryo-EM grid preparation or stored at −80 °C. The presence of all components was indicated by SDS–PAGE and by comparison with MS analysis for *Ct*80S, as done previously^26^.

### Cryo-electron microscopy grid preparation and data collection

Three microliters of *Ct*80S-RAC pullout sample at 200 nM concentration were applied on holey carbon grids (Quantifoil R2/1 grid, Quantifoil Micro Tools) and plunge-frozen into liquid ethane using a Vitrobot environment chamber (FEI). The chamber was programmed to maintain a temperature of 4 °C and 90% humidity. Initial cryo-EM data were collected at the ESRF CM01 and used for sample optimization and grid improvement. Cryo-EM data used for the determination of the structures of *Ct*80S-RAC were collected on an in-house Titan Krios system (FEI) operating at 300 kV using EPU 2 software package. Data were collected on a Quantum-K3 detector using the counting mode. The images were acquired at a nominal magnification of ×81,000, with a total dose of 39.42 e^−^/Å^2^. The defocus range was set from −0.8 to −2.5 and every video was fractioned into 149 frames.

### Single-particle analysis and model building

A total of 6,662 micrographs were used for *Ct*80S-RAC structure determination. The frames were aligned and summed using MotionCor2 whole-image motion correction software^43^. CTFFIND4 was used for contrast transfer function (CTF) estimation of unweighted micrographs^44^. Particle autopicking was performed with Relion 3.1^45^ (Laplacian-of-Gaussian detection) and inspected manually, where majority mis-picked particles or contaminants were removed. Later, particles were extracted (480 × 480 pixels), downsampled (120 × 120 pixels) and subjected to two rounds of reference-free 2D classification in Relion 3.1. The first cycle of 2D classification was performed with a large search range (20 pixels) to achieve the best possible centering of the particles. The second round was performed in higher precision on two times downsampled particles (240 × 240 pixels) with smaller search ranges (5 pixels). Only properly centered class averages were selected for subsequent processing steps. Further processing was performed with cisTEM^46^. The stack of 715,326 particles from 2D classification was imported to cisTEM and auto-refined using a yeast 80S ribosome as reference (low-pass-filtered to 30 Å). Auto-refined particles were subjected to 3D classification, which resulted in removal of ~19% of particles that did not contain RAC. This 3D classification, referred to as focus classification, was performed with the centering of the particles on the exit tunnel. The remaining particles were subjected to additional rounds of 3D focus refinement (focusing on RAC-60S area or RAC-40S area). The refinements were performed with a 60-Å-diameter sphere around the RAC-60S area (Extended Data Fig. 1d, blue sphere) and a 40-Å-diameter sphere around the RAC-40S area (Extended Data Fig. 1d, pink sphere) using the ‘focus_mask’ feature in cisTEM. A more precise masking of RAC was tested, but did not improve the final maps and was not used in the final reconstructions. There were two density maps generated for each RAC conformation, with the primary map considered to be the one refined on the RAC-60S area. The final resolution was measured by Fourier shell correlation (FSC) at the 0.143 value as implemented in cisTEM. The local resolution variations were calculated with ResMap^47^. The 80S ribosome model was refined from the *Ct*80S structure (PDB 7OLC)^26^. As starting models for *Ct*RAC building we used the crystal structures of Ssz1 (PDB 6SR6)^8^ and the components of Zuo1 (PDB 6SR6^8^, 5DJE^22^, 4GMQ^20^ and 2LWX^27^). Because the RAC density was around 4.5–7 Å resolution at the periphery of the map, Ssz1, the Zuo1-N-terminal domain and 4HB were rigid-body-fitted using the crystal or NMR structures. The primary map was used to build all domains of Zuo1, except the 4HB, which had a better visibility in the map refined on the RAC-40S area. Manual building and corrections were done in Coot^48^, and real-space refinement was done in Phenix^49^. Atomic models were validated using Phenix and MolProbity^50^. 3D variability analysis of ribosomal rotation was performed using cryoSPARC (v3.2)^31^ and allowed us to visualize continuous movement of the 40S subunit with respect to 60S.

### Figure preparation

Figures were prepared in GraphPad Prism, PyMOL (The PyMOL Molecular Graphics System, Version 2.0 Schrödinger; LLC), UCSF Chimera^51^ and UCSF ChimeraX^52^.

### Reporting summary

Further information on research design is available in the Nature Portfolio Reporting Summary linked to this Article.

## Online content

Any methods, additional references, Nature Portfolio reporting summaries, source data, extended data, supplementary information, acknowledgements, peer review information; details of author contributions and competing interests; and statements of data and code availability are available at 10.1038/s41594-023-00973-1.

## Supplementary information

Reporting Summary

Supplementary Video 1 **Both RAC conformations on the 80S ribosome can accommodate ribosomal rotation**. The movie is generated by morphing between fully rotated and non-rotated RAC-80S ribosomes (40S is shown in dark gray cartoon; 60S, pale gray; Zuo1, orange; Ssz1, blue). The movie highlights that both RAC conformations (RAC-1 and RAC-2) can accommodate full ribosomal rotation via the Zuo1-MD domain. The movie was generated using PyMOL software.

## Data Availability

EM maps have been deposited in the Electron Microscopy Data Bank under accession codes EMD-14479 for RAC conformation 1 and EMD-14480 for RAC conformation 2. The atomic models have been deposited in the Protein Data Bank under accession nos. 7Z3N and 7Z3O. Source data are provided with this paper.

## Source data

Source Data Extended Data Fig. 1 Unprocessed gel from Extended Data Fig. 1a.
