# Supplementary material for: Structural inventory of cotranslational protein folding by the eukaryotic RAC complex
Source: Nat Struct Mol Biol. 2023 Apr 20;30(5):670–7. doi: 10.1038/s41594-023-00973-1 (PMC10191838; doi:10.1038/s41594-023-00973-1)
Supplement: Supplementary file 1 — Reporting Summary [file 41594_2023_973_MOESM1_ESM.pdf]

nature portfolio

Corresponding author(s): Irmgard Sinning

Last updated by author(s): 26/01/2023

Reporting Summary

Nature Portfolio wishes to improve the reproducibility of the work that we publish. This form provides structure for consistency and transparency in reporting. For further information on Nature Portfolio policies, see our Editorial Policies and the Editorial Policy Checklist.

Statistics

For all statistical analyses, confirm that the following items are present in the figure legend, table legend, main text, or Methods section.

n/a	Confirmed	
☐	☒	The exact sample size (*n*) for each experimental group/condition, given as a discrete number and unit of measurement
☐	☒	A statement on whether measurements were taken from distinct samples or whether the same sample was measured repeatedly
☒	☐	The statistical test(s) used AND whether they are one- or two-sided<br>*Only common tests should be described solely by name; describe more complex techniques in the Methods section.*
☒	☐	A description of all covariates tested
☐	☒	A description of any assumptions or corrections, such as tests of normality and adjustment for multiple comparisons
☒	☐	A full description of the statistical parameters including central tendency (e.g. means) or other basic estimates (e.g. regression coefficient) AND variation (e.g. standard deviation) or associated estimates of uncertainty (e.g. confidence intervals)
☒	☐	For null hypothesis testing, the test statistic (e.g. F, t, r) with confidence intervals, effect sizes, degrees of freedom and P value noted<br>*Give P values as exact values whenever suitable.*
☒	☐	For Bayesian analysis, information on the choice of priors and Markov chain Monte Carlo settings
☒	☐	For hierarchical and complex designs, identification of the appropriate level for tests and full reporting of outcomes
☒	☐	Estimates of effect sizes (e.g. Cohen's *d*, Pearson's *r*), indicating how they were calculated

Our web collection on statistics for biologists contains articles on many of the points above.

Software and code

Policy information about availability of computer code

Data collection	Cryo-EM data was collected using EPU 2 software package.
Data analysis	Cryo-EM images were processed with MotionCor2, CTFFIND4, Relion 3.1, cisTEM 1.0.0, and cryoSPARC V3.2. Molecular models were built with Coot 1.0 and refined using Phenix 1.19. Structures and cryo-EM maps were visualized with PyMOL 2.4.1, UCSF Chimera 1.14 and ChimeraX 1.0 validated with Phenix 1.19 and MolProbity 4.5.

For manuscripts utilizing custom algorithms or software that are central to the research but not yet described in published literature, software must be made available to editors and reviewers. We strongly encourage code deposition in a community repository (e.g. GitHub). See the Nature Portfolio guidelines for submitting code & software for further information.

Data

Policy information about availability of data

All manuscripts must include a data availability statement. This statement should provide the following information, where applicable:

- Accession codes, unique identifiers, or web links for publicly available datasets
- A description of any restrictions on data availability
- For clinical datasets or third party data, please ensure that the statement adheres to our policy

EM maps have been deposited in the Electron Microscopy Data Bank under accession codes EMDB: EMD-14479 for RAC-1 and EMDB: EMD-14480 for RAC-2 conformational states.
The atomic models have been deposited in the Protein Data Bank under accession numbers PDB: 7Z3N and PDB: 7Z3O.

Field-specific reporting

Please select the one below that is the best fit for your research. If you are not sure, read the appropriate sections before making your selection.

☒ Life sciences ☐ Behavioural & social sciences ☐ Ecological, evolutionary & environmental sciences

For a reference copy of the document with all sections, see nature.com/documents/nr-reporting-summary-flat.pdf

Life sciences study design

All studies must disclose on these points even when the disclosure is negative.

Sample size	n=1; We use a unique cryoEM dataset (comprising 6662 micrographs)
Data exclusions	No data were excluded from the analysis.
Replication	n.a., We use a unique cryoEM dataset (comprising 6662 micrographs)
Randomization	n.a. We use a unique cryoEM dataset (comprising 6662 micrographs)
Blinding	n.a.

Reporting for specific materials, systems and methods

We require information from authors about some types of materials, experimental systems and methods used in many studies. Here, indicate whether each material, system or method listed is relevant to your study. If you are not sure if a list item applies to your research, read the appropriate section before selecting a response.

Materials & experimental systems

n/a	Involved in the study	
☐	☒	Antibodies
☐	☒	Eukaryotic cell lines
☒	☐	Palaeontology and archaeology
☒	☐	Animals and other organisms
☒	☐	Human research participants
☒	☐	Clinical data
☒	☐	Dual use research of concern

Methods

n/a	Involved in the study	
☒	☐	ChIP-seq
☒	☐	Flow cytometry
☒	☐	MRI-based neuroimaging

Antibodies

Antibodies used	PAP (Sigma-Aldrich, P1291) antibody
Validation	Sigma Aldrich standard validation protocol.

Eukaryotic cell lines

Policy information about cell lines

Cell line source(s)	German collection of microorganisms and cell cultures (DSMZ)
Authentication	1495 (wild-type) Chaetomium thermophilum
Mycoplasma contamination	tested negative
Commonly misidentified lines (See ICLAC register)	n.a.
