# Supplementary figures and images for: Structural inventory of cotranslational protein folding by the eukaryotic RAC complex
Source: Nat Struct Mol Biol. 2023 Apr 20;30(5):670–7. doi: 10.1038/s41594-023-00973-1 (PMC10191838; doi:10.1038/s41594-023-00973-1)

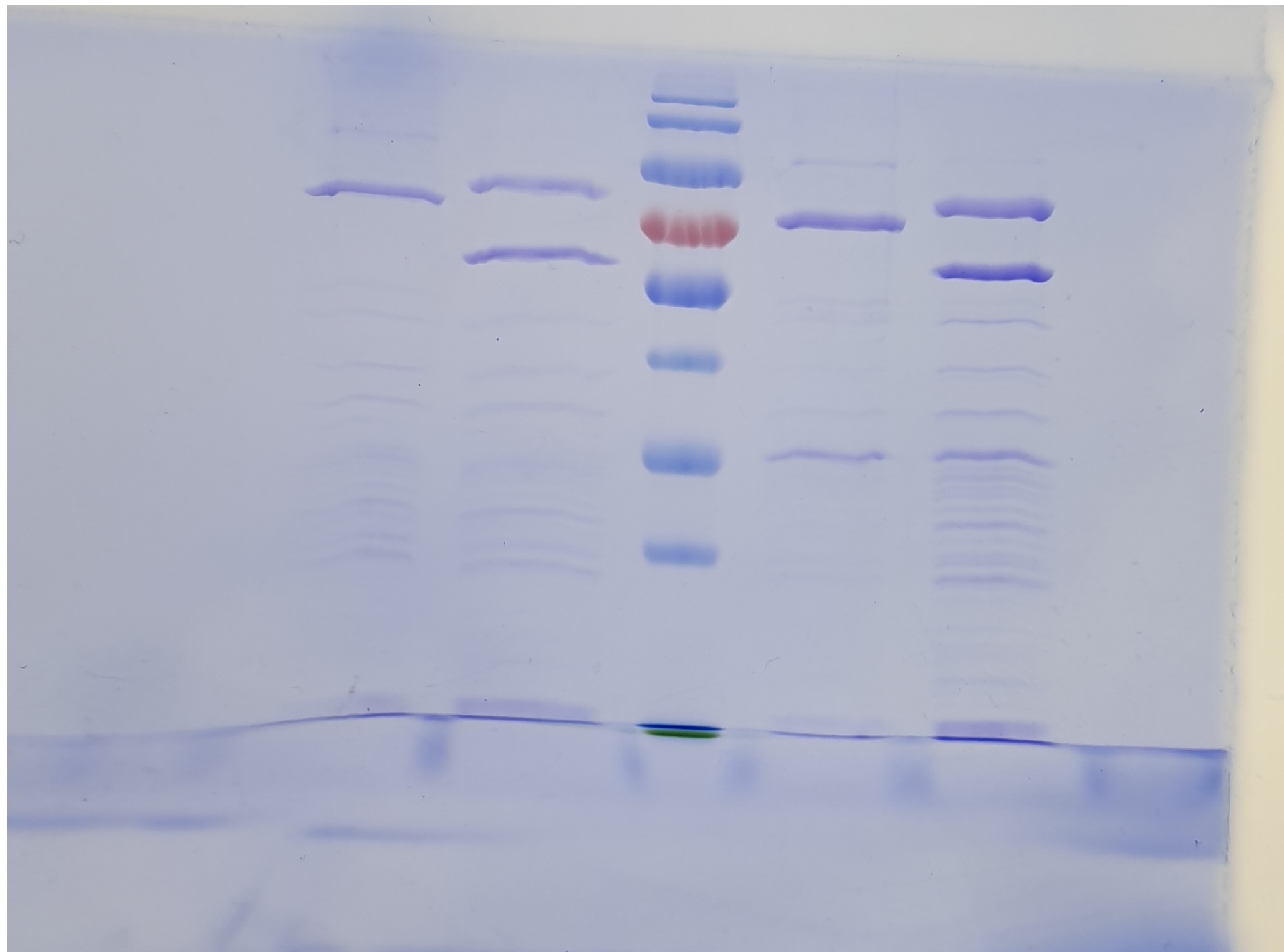

Supplement: Source Data Extended Data Fig. 1 — Unprocessed gel from Extended Data Fig. 1a. [file 41594_2023_973_MOESM3_ESM.pdf]
